# Physicochemical Characterization and Thermodynamic Studies of Nanoemulsion-Based Transdermal Delivery System for Fullerene

**DOI:** 10.1155/2014/219035

**Published:** 2014-08-03

**Authors:** Cheng Loong Ngan, Mahiran Basri, Minaketan Tripathy, Roghayeh Abedi Karjiban, Emilia Abdul-Malek

**Affiliations:** ^1^Department of Chemistry, Faculty of Science, Universiti Putra Malaysia, 43400 Serdang, Selangor, Malaysia; ^2^Halal Products Research Institute, Universiti Putra Malaysia, 43400 Serdang, Selangor, Malaysia; ^3^Laboratory of Fundamentals of Pharmaceutics, Faculty of Pharmacy, Universiti Teknologi MARA (UiTM), Puncak Alam Campus, 42300 Selangor, Malaysia; ^4^Brain and Neuroscience Communities of Research, Universiti Teknologi MARA (UiTM), 40450 Shah Alam, Selangor, Malaysia

## Abstract

Fullerene nanoemulsions were formulated in palm kernel oil esters stabilized by low amount of mixed nonionic surfactants. Pseudoternary phase diagrams were established in the colloidal system of PKOEs/Tween 80 : Span 80/water incorporated with fullerene as antioxidant. Preformulation was subjected to combination of high and low energy emulsification methods and the physicochemical characteristics of fullerene nanoemulsions were analyzed using electroacoustic spectrometer. Oil-in-water (O/W) nanoemulsions with particle sizes in the range of 70–160 nm were formed. The rheological characteristics of colloidal systems exhibited shear thinning behavior which fitted well into the power law model. The effect of xanthan gum (0.2–1.0%, w/w) and beeswax (1–3%, w/w) in the estimation of thermodynamics was further studied. From the energetic parameters calculated for the viscous flow, a moderate energy barrier for transport process was observed. Thermodynamic study showed that the enthalpy was positive in all xanthan gum and beeswax concentrations indicating that the formation of nanoemulsions could be endothermic in nature. Fullerene nanoemulsions with 0.6% or higher xanthan gum content were found to be stable against creaming and flocculation when exposed to extreme environmental conditions.

## 1. Introduction 

Nanoemulsions are non-equilibrium colloidal systems formed by forcing two immiscible liquids into homogeneous state which is kinetically stable with particle size ranging from 20 to 200 nm [1]. Unlike microemulsions, both systems can be distinguished in terms of their thermodynamic stabilities where microemulsions are thermodynamically stable, whereas nanoemulsions are thermodynamically unstable. The use of nanoemulsions in efficient delivery of active ingredients or drugs into human body has been widely investigated. There are many areas such as pharmacy, cosmetics, and food technologies as well as non-medical-related products such as polymers and pesticides which utilize the concept of nanoemulsification. In fact, most of the drugs are lipophilic in nature [2], and oil-in-water (O/W) type of emulsion is widely developed over the water-in-oil (W/O) emulsion where oil acts as carrier for drugs.

In drug delivery system, toxicity of the final product depends on the ingredients which constitute the emulsion system. Microemulsions have a presence of noxious cosurfactant and higher concentration of surfactant that leads to unwanted toxicity, whereas nanoemulsions are devoid of such disadvantage. Nonionic surfactants have prominent advantages as compared to anionic or cationic surfactants in regard to lesser toxicity and biodegradability [3, 4]. Previously, several studies on the thermodynamics of microemulsions with different mixed surfactant ratios and types of cosurfactant were reported [5, 6]. A thorough literature study showed that limited attempts have been made with regard to the thermodynamics of nanocolloidal systems in the presence of rheological modifiers like hydrocolloid gums.

The present study intends to develop a novel nanoemulsion for transdermal application using palm kernel oil esters as biocompatible carrier for fullerene. The core of this study is based on characterization of physicochemical behavior of the developed system in emphasizing the involved thermodynamic. Fullerene is a hydrophobic molecule composed entirely of carbon atoms which are interconnected to each other via single and double bonds forming a spherical shape [7]. Large production of fullerene via Krätschmer-Huffman arc process [8] and discovery of its indication as a powerful/potential antioxidant have attracted many investigations [9–11].

There have been a number of reports indicating the utility of fullerene and its derivatives in oxidative stress-related diseases, such as HIV, Parkinson's disease, and allergy [12–14], as well as in cosmetics [15–17], with no acute or subacute toxicity to human body [18–21]. The excellent antioxidant property of fullerene may be linked to its capability to break the C=C bonds, thereby yielding the corresponding radical adducts [9, 10], or by catalytic dismutation [22]. Besides medicinal applications, fullerenes are also used in DNA photocleavage, enzyme inhibition, and antimicrobial activity and as contrast agent or radiotracer [23]. The primary advantages of transdermal antioxidant using fullerene incorporated nanoemulsions could be of a direct application in protecting the human skin that is exposed to ultraviolet (UV) radiation. Potential skin irritation can be avoided due to the presence of lower concentration of surfactants as compared to that in microemulsions.

In this study, the physical and structural aspects of fullerene nanoemulsions comprised of mixed nonionic surfactants and palm kernel oil esters which are presented here. Different formulations of fullerene stabilized by xanthan gum were prepared by using combination of high and low energy emulsification techniques. The physicochemical properties of the novel formulations were characterized with an emphasis on the thermodynamic and flow behavior.

## 2. Materials and Methods

### 2.1. Materials

Powdered fullerene (C_60_) with 99.5% purity, xanthan gum from* Xanthomonas campestris*, white beeswax, polyoxyethylene sorbitan monooleate (Tween 80), and sorbitan monooleate (Span 80) were purchased from Sigma-Aldrich (St. Louis, USA). The average molecular weight of xanthan gum was estimated as 152 kDa [24]. Palm kernel oil esters (PKOEs) were synthesized in our laboratory through enzymatic transesterification of palm kernel oil and oleyl alcohol using Lipozyme RM IM as the catalyst [25]. The fatty acid composition of PKOEs is shown in Table 1. Preservative, phenonip, was obtained from Bramble Berry (Bellingham, USA). Deionized water was purified using Milli-Q water system (Billerica, USA).

### 2.2. Construction of Pseudoternary Phase Diagrams

Fullerenes were assayed at 160 *μ*g/g of PKOEs by stirring using magnetic stirrer at 400 rpm for 24 h at room temperature (298.0 ± 0.5 K). Mixture of fullerene solution with Tween 80 was filled in 11 screw-cap vials at different ratios (w/w) of 10 : 0, 9 : 1, 8 : 2, 7 : 3, 6 : 4, 5 : 5, 4 : 6, 3 : 7, 2 : 8, 1 : 9, and 0 : 10, respectively. Deionized water 5% (w/w) was added each time into the vials containing mixtures. All components were weighed using analytical balance (Dragon 204, Mettler Toledo, Spain) and microbalance (CPA2P, Sartorius, Germany). Cap was tightened and homogenized for 20 min using a vortex mixer equipped with flask accessory (VTX-3000L, Uzusio, Japan). Subsequently, the samples were centrifuged (Hermle Labor Technik, Germany) at 4000 rpm for 15 min to observe any phase separation through cross polarized plates for visual identification of physical appearances. The above steps were repeated with consecutive addition of 5% (w/w) deionized water.

The phase behavior study for mixed surfactant system (Tween 80 : Span 80) was conducted. The pseudoternary phase diagram was constructed at room temperature (298.0 ± 0.5 K) and was plotted using SigmaPlot version 12 by Systat Software Inc. (San Jose, USA). Classification of phase state was made according to the physical appearance observed which was categorized into isotropic, homogeneous, and two or multiphase regions.

### 2.3. Hydrophilic-Lipophilic Balance (HLB)

An approach using HLB concept, an empirical parameter, was applied to determine the hydrophilic and hydrophobic content of surfactants and was used to study the phase behavior of the mixtures. This method aims to achieve the required HLB value for a given oil to obtain stable emulsion system. High HLB value indicates high hydrophilicity of the surfactant system or vice versa. The HLB values of mixed binary surfactant systems were calculated by Griffin's formula which was applicable to nonionic surfactants as shown in [26]

(1) $$\begin{array}{c} {\text{HLB}}_{\text{mix}}\text{ = }\frac{\text{wt.\% of surfactant }A}{\text{100}}\times \text{(}{\text{HLB}}_{A}-{\text{HLB}}_{B}\text{) }+{\text{HLB}}_{B}. \end{array}$$

### 2.4. Selection of Preformulation Concentrates

Preformulation (Pre-F) was selected from the pseudoternary phase diagrams based on the following:

- low percentage of surfactant to avoid potential adverse toxicology and dermatology effects [27];
- appreciable percentage of dispersed phase (oil phase) to favor the formation of O/W emulsion system which gained the consumer acceptance for most skin care products.

### 2.5. Preparation of Fullerene Nanoemulsions (FNEs)

Selected Pre-F was prepared using high energy followed by low energy emulsification technique. Formulations with xanthan gum at varying concentrations of 0.2, 0.4, 0.6, 0.8, and 1.0% (w/w) and beeswax concentrations of 1, 2, and 3% (w/w) were prepared. 0.7% (w/w) of phenonip was added into the formulations as antimicrobial agent. Nanoemulsions were prepared by stirring oil and aqueous phase separately at 400 rpm at 373 K to fully dissolve all the ingredients. The oil phase was added slowly into the aqueous phase and homogenized at 4,000 rpm for 15 min using a high shear homogenizer (PT3100, Kinematica AG, Switzerland). The emulsions formed were then cooled down to room temperature (298 ± 2 K) while stirring at 200 rpm using an overhead stirrer (RW20 digital, IKA-Werke, Germany).

### 2.6. Acoustic and Electroacoustic Spectroscopy

The FNEs mean droplet size was measured using acoustic and electroacoustic spectrometer (DT-1200, Dispersion Technology, USA) equipped with electroacoustic probes and a stirrer. It allows the determination of the distribution and the droplet size down to the nanometer range. During the measurement process, parameters such as ultrasonic attenuation, sound velocity, and acoustic impedance could be determined at different frequencies. The mathematical modeling of these measurements results in the calculation of the droplet size. With electroacoustic spectroscopy, it is unnecessary to carry out sample dilution which allows the characterization of the real concentrated dispersions. Sample dilution will alter the overall system properties of the emulsion due to the changes in actual percentage composition of each component. 100 mL of FNEs was loaded into the spectrometer chamber. The parameters of Debye length and dynamic mobility of the FNEs droplets were also determined with the software provided by feeding the respective conductance data.

### 2.7. Characterization Studies of FNEs

The viscosity of colloidal systems was measured using a dynamic shear rheometer (Kinexus rotational rheometer, Malvern Instrument, UK) at a fixed shear rate of 0.1 s^−1^ and at 4 different temperatures (298, 303, 308, and 313 K). The measurements were performed within 24 h after sample preparation using cone and plate geometry (4°/40 mm), the gap being set at 0.15 mm. Samples were loaded and left for 5 min to equilibrate, before carrying out the measurement. Viscosity of each sample was recorded in Pa s at an interval of 5 s till 60 data points are generated and the average viscosity value was calculated. Low viscosity samples were measured using a viscometer (LV, Brookfield, USA) equipped with plate geometry (CP43) at a controlled shear rate of 100 rpm. The gap between the cone and the plate geometry was 0.02 mm. Viscosities of each sample were recorded at an interval of 15 s till 20 data points are generated and the average viscosity was taken as the viscosity of the compositions. In order to avoid any instrumental error, calibration was carried out first by measuring the viscosity of water.

The rheological behavior of fullerene-based colloidal systems was examined at varying controlled shear rate mode from 0.1 to 100 s^−1^ at 298 K. FNEs were subjected to centrifugation at 2000 rpm for 2 min to get rid of any air pockets present within the emulsion before loading onto the plate. The rheograms obtained were analyzed using power law model:

(2) $$\begin{array}{c} \sigma =K\cdot \gamma^{n}, \end{array}$$

where *γ* is the shear rate (s^−1^), *n* is the flow behavior index (dimensionless), *σ* is the shear stress (Pa), and *K* is consistency index (Pa s^*n*^).

Densities of FNEs were measured at four different temperatures (298.15, 303.15, 308.15, and 313.15 K) using a densitometer (DM2911, Rudolph Analytical Research, USA). Sample was injected slowly into the valve where the sample moves through the glass tube to ensure that no air pocket was trapped along the tube. The densitometer was calibrated by using pure water and dry air. The densities have precision <±10^−5^  kgm^−3^. The pH values of FNEs were measured using a pH meter (S20-SevenEasy, Mettler Toledo, Switzerland) and the conductivities of FNEs were measured using a conductivity meter (SG3-SevenGo, Mettler Toledo, Switzerland). Calibration was carried out using different pH buffer solutions (pH 4, 7, and 10) and conductivity standards (1413 *μ*S/cm and 12.88 mS/cm) provided by the manufacturer, respectively. To obtain a representative sample, 20 strokes were performed to expose the probe to sample before running the measurement. All measurements were carried out repetitively five times at 298 K and the measurements were reported as the average value of five readings.

### 2.8. Accelerated Stability Study

An accelerated storage testing was carried out to predict the long-term physical stability of the nanoemulsions. Samples were subjected to centrifugal force of 4000 rpm for 15 min. In the thermal stress test, FNEs were kept at storage temperatures of 298 K and 318 K for 90 days. FNEs were also tested for freeze-thaw cycles by adding 5 mL of samples into thin-walled glass tubes which were allowed to freeze at 269 K for 24 h and thawed at room temperature for another 24 h. The above step was repeated for three cycles. Visual assessment under cross polarized plates was done to evaluate the physical appearance of the samples.

## 3. Results and Discussion

### 3.1. Phase Behavior of PKOEs: Fullerene/Surfactant(s)/Water System

**Figure 1:**
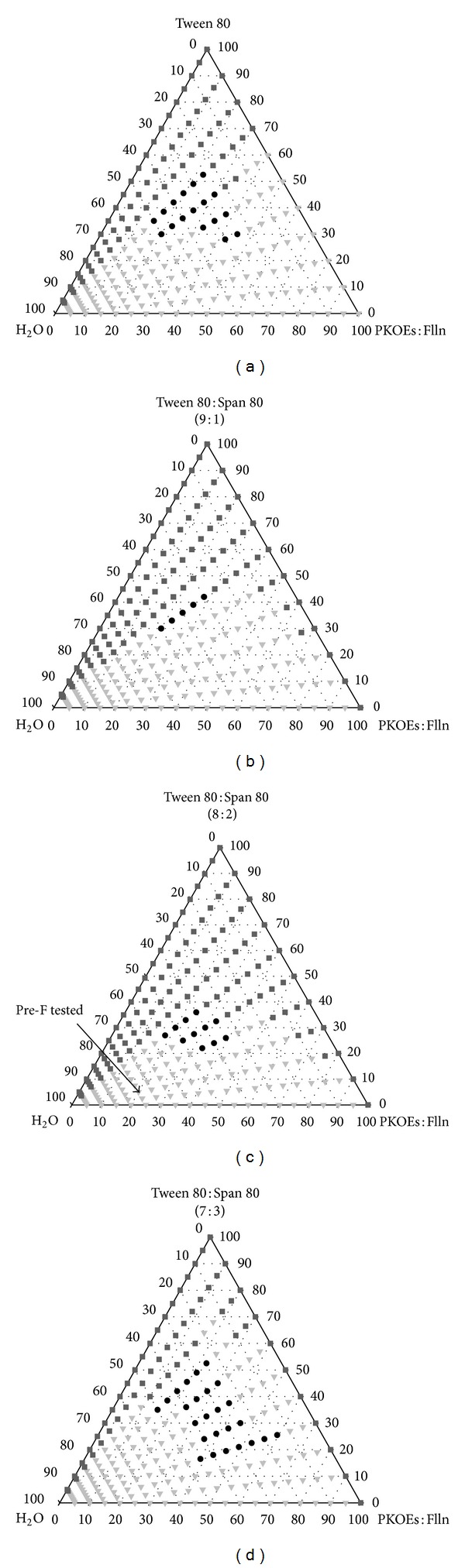
Pseudoternary phase diagram of PKOEs : fullerene (Flln)/Tween 80 : Span 80/Water in the MSRs of (a) 10 : 0, (b) 9 : 1, (c) 8 : 2, and (d) 7 : 3 at 298 K. ■ = Isotropic; ● = Homogeneous; ▼ = Two-phase.

The pseudoternary phase diagram was constructed on the basis of initial experiments to show the relationship between composition involving PKOEs : fullerene, single surfactant (Tween 80), and water using minimum energy through vortexing. The phase-forming behavior of the PKOEs : fullerene/Tween 80/water system at 298 K is presented in Figure 1(a). Classification of regions was made based on optical visualization through cross polarized plates after centrifugation process. The appearance of single phase lies in the isotropic region with transparent monophasic liquid whereas the homogeneous region was identified as milky/cloudy white liquid. The remaining region represents the two-phase region which has two distinct layers of liquid/gel phases. It was observed that oil : surfactant ratio at 10 : 0, 9 : 1, and 8 : 2 at all water percentages showed isotropic system but started to exhibit homogeneous system at ratio of 7 : 3. Nonetheless, the application of the single surfactant did not produce large enough monophasic region.

Mixture of surfactants was used instead of a single surfactant. This combination of surfactants exhibited better performance in many industrial applications [28, 29]. Combination of Tween 80 and Span 80 has high compatibility to build a stable interfacial film and give better performance in emulsification process in most of the studies [30–32]. Small amount of Span 80 assisted in reducing the interfacial tension between oil and water and in acquiring tighter molecular packing between the surfactant molecules at the oil-water interface. The phase-forming behavior of the PKOEs : fullerene/Tween 80 : Span 80/water system at three different mixed surfactant ratios (MSRs) of 9 : 1, 8 : 2, and 7 : 3 at 298 K is presented in Figures 1(b), 1(c), and 1(d), respectively.

The HLB values of single and binary surfactant systems of Tween 80 and Span 80 relating to the formation of monophasic region in the pseudoternary phase diagram are shown in Table 2. The increasing of Span 80 content in the mixed surfactant system at MSR from 9 : 1 to 8 : 2 increased the monophasic region but it was reduced dramatically at MSR of 7 : 3. The largest monophasic region formation was noticed using mixed surfactant system Tween 80 : Span 80 (MSR 8 : 2) with HLB of 12.86. The optimized HLB value obtained from mixed surfactants displayed that the enhanced flexibility of surfactant layer is able to partition itself efficiently at the oil-water interface. Mixed surfactants were found to increase the monophasic region where it shifted towards the oil-rich corner of the phase diagrams. This indicated that higher oil concentration was incorporated within the system than the single surfactant did. The synergistic effect of the surfactant molecules interaction was due to the mixed micelles formation which enhanced the solubilization capacity, with the increase in hydrophobicity of the binary surfactant systems [33].

As seen in Figure 1(c), the isotropic phases were located along the oil-surfactant axes at any ratio indicating the total solubility between the two components which was consistent with previous studies [31, 34]. Without the presence of water, oil phase was able to merge completely into the surfactant with the lipophilic tail of surfactant molecules arranging themselves towards the oil phase. The large isotropic region which appeared at the rich surfactant area allowed the formation of micellar solution or water-in-oil (W/O) microemulsion. As the dominant species are mainly surfactant molecules, relative amount of oil phase is rearranged within the surfactant vesicles/layers where the hydrophilic head usually surrounds the oil phase. When the total amount of surfactant used decreased to less than 40% (w/w) in the system, transition took place from isotropic to homogeneous state immediately. The oil phase started to break into individual droplets where water started to take over to become the continuous phase. The system evolved from water-in-oil (W/O) to an oil-in-water (O/W) structure whereby, in the homogeneous state, it showed lower stability than the isotropic due to its polydispersity. The phase equilibrium for two-phase region was not determined. Based on the results and reference [35], a Pre-F composed of 20% PKOEs : fullerene, 5% mixed surfactants (i.e. Tween 80 : Span 80, 8 : 2), and 75% water was chosen for further studies.

### 3.2. Characterization of FNEs

The mean droplet size of FNEs with different components concentration ranged from 70 to 160 nm with fitting error less than 5% as tabulated in Table 3. According to Dukhin and Fairhurst, the emulsion comprises primarily of very small droplet size as large droplets are broken down due to high shear homogenisation and with a fraction of larger droplets indicating smaller droplets are coalescing [36]. By using acoustic measurements, dynamic changes in the state of an emulsion can be followed in real time. It was observed that the droplet size increased with the increase of xanthan gum concentration to 0.4% (w/w) but started to maintain its size around 100 to 130 nm at higher concentration. These results indicated that concentration of 0.4% (w/w) xanthan gum and below was not able to establish secondary layer to completely surround the emulsion droplets; instead it induced the fusion of emulsion droplets due to depletion forces [37]. However, formulations with higher xanthan gum concentration were able to keep the droplets away from each other by forming steric stabilizing polymer-coated droplets due to the sufficient polymer chain to cover the surface of oil droplets [38]. This suggests that a minimum xanthan gum concentration is needed to form a polymer network that is capable of maintaining the droplets apart [39, 40]. Pre-F and FNE1 showed the smallest droplet size because they exhibit perfect collision during Brownian motion for limited period of time. In the absence of xanthan gum, the emulsification process will not be affected by the restriction of the high viscosity xanthan gum; thus no smaller droplet will be observed.

**Figure 2:**
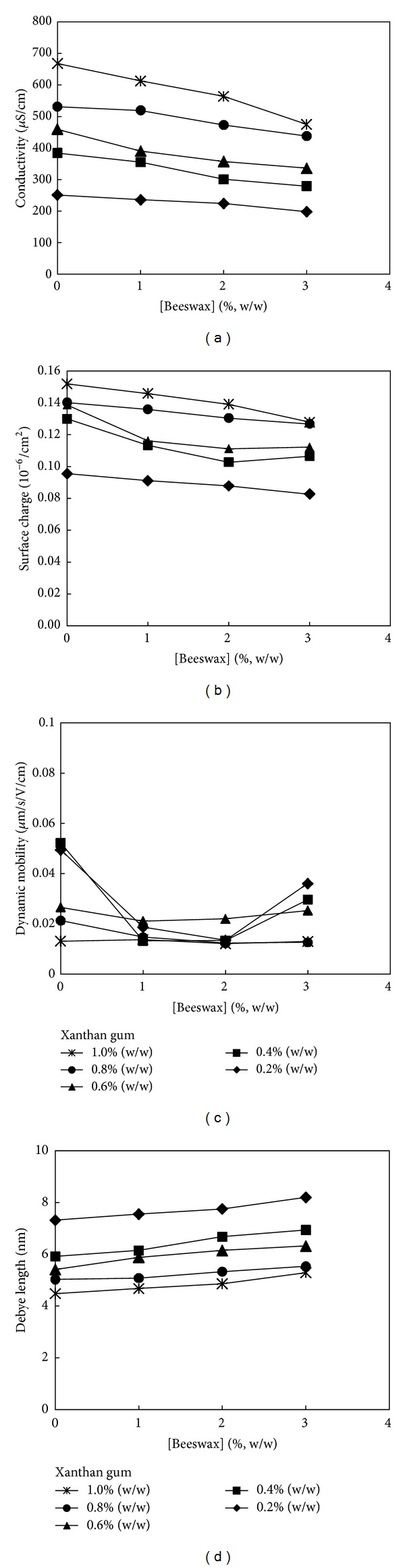
Effects of beeswax and xanthan gum concentration on (a) electrical conductivity, (b) surface charge, (c) dynamic mobility, and (d) Debye length of FNEs.

The pH of a healthy human skin on average is 5.5 [41]. In Table 3, the results showed that all formulations were compatible with human skin with ±1.5 variation in pH which is suitable for human application without affecting the natural pH of human skin. In Figure 2(a), the electrical conductivity of FNEs was observed to increase with increasing xanthan gum concentration but decrease with increasing beeswax concentration. A similar observation was reported by a previous study [42]. With the absence of xanthan gum and beeswax in the colloidal systems, the electrical conductivity of Pre-F and FNE1 measured is 55.4 and 63.8 *μ*S/cm. A sharp increase in electrical conductivity is observed with the incorporation of xanthan gum into the system and this may be due to the nature of xanthan gum as an anionic polysaccharide. The effect of xanthan gum and beeswax concentration on surface charge is also depicted in Figure 2(b). In terms of surface charge of emulsion droplets, the surface charge increases with increasing xanthan gum concentration but decreases slowly with increasing beeswax concentration.

The electrical conductivity of the FNEs is also related to the ability of emulsion droplets to conduct an electric current which correlates to their mobility. The dynamic mobility of the colloidal systems is presented in Figure 2(c). It is evident from Figure 2(c) that the dynamic mobility of the colloidal systems decreases with the increase in concentration of xanthan gum. However, the addition of beeswax decreases the mobility of the oil droplet. The increase in beeswax concentration increases the mobility of the oil droplet. The Debye length of the colloidal systems ranging from 4.48 to 8.20 nm is plotted in Figure 2(d). Debye length for Pre-F and FNE1 was 15.56 and 14.52 nm, respectively. With the introduction of xanthan gum into the colloidal system, the Debye length reduced drastically. The Debye length is found to increase with increasing the concentration of both xanthan gum and beeswax. These findings point to the fact that xanthan gum and beeswax can work efficiently as viscosity enhancers through controlling the Brownian motion only above certain concentration. Further, the observations related to dynamic mobility and that of the Debye length are complementing each other in this case.

### 3.3. Shear Viscosity of the FNEs

**Figure 3:**
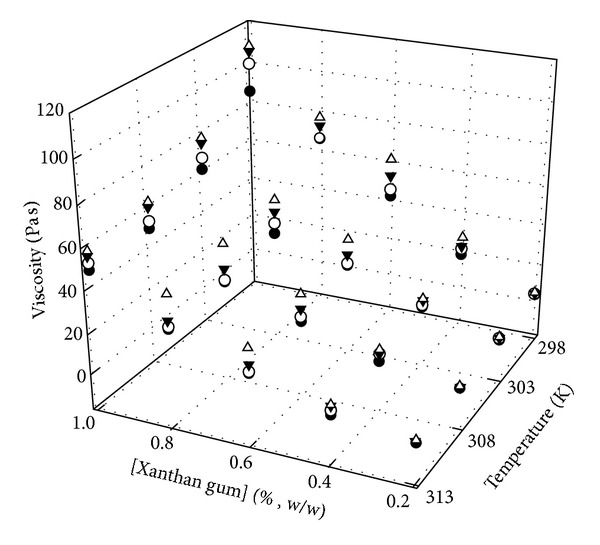
Viscosities of Pre-F and FNEs at different temperatures with varying concentration of xanthan gum containing 0% (●), 1% (○), 2% (▼), and 3% (Δ) of beeswax.

The shear viscosities, *η*, of FNEs were measured at controlled shear rate at four different temperatures, 298, 303, 308, and 313 K. The shear rate that was chosen to be operated on the rheometer/viscometer gave the maximum percentage of torque indicating the high accuracy of reading. In Figure 3, the viscosities of FNEs increased with the increase in concentration of xanthan gum and beeswax. Nevertheless, the viscosities decreased with the increase in temperature. The increase in temperature enhances the thermal energy of the system, thereby causing the oil droplet to vibrate and travel faster in Brownian motion within the aqueous medium, thus easier for the emulsion to flow. Based on Arrhenius equation, there is an inverse relationship between the viscosity and the temperature [43]:

(3) $$\begin{array}{c} \eta =\eta_{o}\exp\left(\frac{E_{a}}{RT}\right), \end{array}$$

where *η*_*o*_ is a constant, *E*_*a*_ is the activation energy, *T* is the absolute temperature, and *R* is the gas constant (8.314 KJ mol^−1^). Figure 3 shows that the viscosities of the formulations decrease with increase in temperature within the systems which was found to be adequately described by the Arrhenius equation. Another reason can be also related to the loose packing between polymer chains which can cause more space for the polymer chain to slip through.

**Figure 4:**
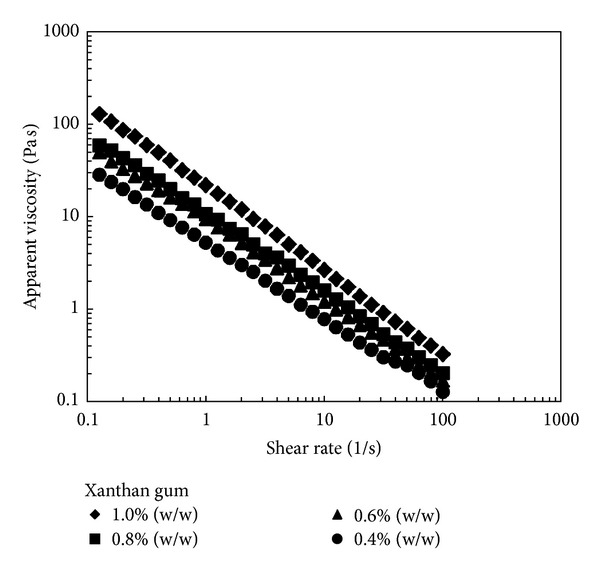
Shear rate dependence of steady shear viscosity for FNEs with 1% (w/w) of beeswax with different xanthan gum concentrations.

Steady shear flow properties of FNEs with different compositions of xanthan gum and beeswax concentrations were measured over a range of shear rates. The flow behaviors of FNEs are particularly important to predict the end-product's texture attribute and to ease the industrial handling processes such as flow pump and agitation tank [43].  Figure 4 shows the shear rate dependence of the apparent viscosity for FNEs with 0.4, 0.6, 0.8, and 1.0% (w/w) xanthan gum. The flow behavior of FNEs could be well described by the power law model. As the flow behavior index, *n*, gets less than 1, the more pronounced is the shear thinning behavior (pseudoplastic), which exhibits non-Newtonian behavior [44].  Table 4 shows the values of power law parameters obtained by linear regression analysis. The data exhibited *n* values less than 0.3 with *R* ^2^ values higher than 0.970 in all FNEs. The consistency index was also increased with the increase of xanthan gum concentration demonstrating the strengthening in structure formation of nanoemulsion systems [45]. It was clearly shown that the apparent viscosity decreased as the shear rate was increased for all FNEs due to the breakdown of colloidal structure [46]. Initially, when low shear rate was applied, xanthan gum which was composed of large molecules formed a polymer network structure surrounding the oil droplets through hydrogen bonding between xanthan gum molecules, thus resulting in high shear viscosity of FNEs. It was reported that, upon application of high shear rate, the flow of the emulsion droplets tended to align with the direction of the shear flow, hence disentangling the polymer network and reducing the apparent viscosity of the samples [45].

The activation enthalpy, ΔH*, for the viscous flow of the investigated nanocolloidal systems was estimated according to the following equation:

(4) $$\begin{array}{c} \eta =\left(\frac{Nh}{\upsilon }\right)\exp\left(\frac{{\Delta H}_{\text{vis}}^{*}}{RT}\right)\exp\left(\frac{{-\Delta S}_{\text{vis}}^{*}}{R}\right), \end{array}$$

or

(5) $$\begin{array}{c} \ln\eta =\left[\ln\left(\frac{Nh}{\upsilon }\right)-\frac{{\Delta S}_{\text{vis}}^{*}}{R}\right]+\frac{{\Delta H}_{\text{vis}}^{*}}{RT}, \end{array}$$

where *h*, *N*, *υ*, and Δ*S*_vis_* are Plank's constant, Avogadro's number, molar volume, and entropy of activation, respectively. In (5), the first term in the right hand side (in the bracket) has a constant value. So, the values of Δ*H*_vis_* can be determined from the slope of the nonlinear plot of ln *η* versus *T*. Hence, the relationship between ln *η* and *T* can be expressed in terms of a polynomial as follows:

(6) $$\begin{array}{c} \ln\eta =a+bT+{cT}^{2}, \end{array}$$

and the values of Δ*H*_vis_* can be obtained from its derivative with respect to *T* followed by

(7) $$\begin{array}{c} \frac{d\ln\eta }{dT}=\frac{{-\Delta H}_{\text{vis}}^{*}}{{RT}^{2}}=b+2cT, \end{array}$$

where *a*, *b*, and *c* are the fitting parameters, obtained by the computational method.

The values of molar volume were kept constant as the experimental densities of the nanocolloidal systems showed very insignificant dependence over temperature. Furthermore, the values of Δ*H*_vis_* were used to calculate the entropy of activation followed by Gibbs free energy equation:

(8) $$\begin{array}{c} {\Delta S}_{\text{vis}}^{*}=\frac{\left({\Delta H}_{\text{vis}}^{*}-{\Delta G}_{\text{vis}}^{*}\right)}{T}, \end{array}$$

where the values of Δ*G*_vis_* were estimated by

(9) $$\begin{array}{c} {\Delta G}_{\text{vis}}^{*}=RT\ln\left(\frac{\eta \upsilon }{Nh}\right). \end{array}$$

The activation parameters for FNEs with different compositions are tabulated in Table 5. Δ*G*_vis_* and Δ*H*_vis_* values were positive in most formulations which was similar to other transport processes [5]. It is seen that the addition of xanthan gum resulted in a longitudinal decrease in the values of enthalpy whereas the reverse was the case with the increase in temperature. The positive Δ*S*_vis_* value with increasing xanthan gum concentration for all compositions indicated that more polymer chains were crowded at the interface of the emulsion droplet, thus suggesting structure enhancement of the emulsion. FNEs composed of 1.0% xanthan gum behaved in an order system by showing lowest Δ*S*_vis_* which form a colloidal network spreading through the entire system, which contribute to the improved stability. However, in all cases, the Δ*S*_vis_* values were gradually increased with the increase of temperature indicating that microscopic droplets scatter much frequently creating disorder arrangement in the colloidal system due to the increase in kinetic energy.

### 3.4. Accelerated Stability Study

Assessment of long-term stability of FNEs shelf life under environmental storage conditions can be very tedious and time consuming which is considered uneconomical. The development of products ought to be fast-paced yet reliable to meet the demand of the consumers. Thus, FNEs were let to experience a variation of extreme storage conditions to predict the samples' ability to withstand over a period of time. Centrifugation can accelerate the rate of creaming or sedimentation which demonstrates that the breakdown of an emulsion can be related to the action of gravitational force. O/W emulsion system often exhibits creaming rather than sedimentation due to the lower density of the oil droplet compared to the aqueous medium. The result of the accelerated stability study of FNEs is shown in Table 6. Changes of physical appearance were also observed upon completion of the centrifugation process. Pre-F and FNE1 were found to be separated into two distinct layers (oil layer: top; aqueous layer: bottom) upon centrifugation resulting from instability in the emulsion system. Absence of xanthan gum as a viscosity enhancer (thickener) in the continuous phase caused failure in resisting the movement of the oil droplet against the gravitational force.

Sample storage at elevated temperature contributed to the higher kinetic energy in the Brownian motion of the oil droplets. This is possibly to speed up the movement and more collision between the oil droplets at higher temperature. Only FNEs which were stable against centrifugation were tested on storage at elevated temperature. Our results showed that FNEs with more than 0.6% xanthan gum (w/w) were able to maintain the homogeneity of the emulsion only up to 90 days. The phase separation decreases with the increase in xanthan gum concentration possibly due to the formation of more ordered polymer structure formed within the emulsion system.

In the freeze-thaw cycle, FNEs, except Pre-F and FNE1, were found to be stable by maintaining their homogenous state. On freezing the samples, oil droplets segregated themselves from the emulsion by the crystallized ice particle resulting in the disruption of lipid film surrounding the droplets. When the samples were thawed, the droplets melted and immediately coalesced between approaching droplets, resulting in oiling off from the emulsions which was indicated by phase separation. However, FNEs with xanthan gum maintained their homogeneity whereas Pre-F and FNE1 (without xanthan gum) exhibited phase separation. Xanthan gum being dissolved in the water reduced the reassociation of oil droplets and that phenomenon in the damaged network led to less syneresis [47]. Xanthan gum also reduced the formation of ice crystals. Hence, FNEs containing xanthan gum were suitable to be stored at freezing temperature.

## 4. Conclusions

Pseudoternary phase diagrams served as template to obtain emulsion-based preformulation and were further developed into nanoemulsions with small particle size (<200 nm) by high-low energy emulsification method. Mixed surfactant systems demonstrated better performance in obtaining larger monophasic zones compared to single surfactant system. The relationship between viscosity and temperature of the fullerene nanoemulsions agreed well with Arrhenius equation at all tested temperatures of 298–313 K. In the presence of xanthan gum, fullerene nanoemulsions exhibited pseudoplastic behavior. The activation enthalpy for viscous flow (Δ*H*_vis_*) was found to be composition dependent and the Gibbs free energy of fullerene nanoemulsions containing xanthan gum and beeswax was more positive than the preformulation. The stability of fullerene nanoemulsions was xanthan gum content dependent which remained stable up to 90 days of storage period at elevated temperature. This methodology can be used to understand and assess the structural formation in the development of a highly stable nanoemulsion for transdermal delivery.

## Figures and Tables

**Table 1:** The percentage of fatty acid composition in palm kernel oil esters.

Palm kernel oil esters composition	(number of carbon atoms : number of double bonds)	Percentage (%)
Oleyl caproate	C24 : 1	0.5
Oleyl caprate	C26 : 1	5.6
Oleyl caprylate	C28 : 1	5.9
Oleyl laurate	C30 : 1	54.1
Oleyl myristate	C32 : 1	13.9
Oleyl palmitate	C34 : 1	6.2
Oleyl stearate	C36 : 1	1.2
Oleyl oleate	C36 : 2	6.4
Oleyl linoleate	C36 : 3	1.7

**Table 2:** Calculated hydrophilic-lipophilic balance (HLB) values of the nonionic surfactants in the single and binary surfactant systems.

Surfactant/mixed surfactant	HLB number
Tween 80	15.00
Span 80	4.30
Tween 80 : Span 80 (9 : 1)	13.93
Tween 80 : Span 80 (8 : 2)	12.86
Tween 80 : Span 80 (7 : 3)	11.79

**Table 3:** Composition and physicochemical characteristics of FNE formulations.

Formulation	Components concentration (%, w/w)							Diameter (±standard deviation) (nm)	pH
	PKOEs	Tween 80	Span 80	Phenonip	Xanthan gum	Beeswax	Water		
Pre-F	20	4	1	0	0	0	75.0	89.7 ± 0.10	5.70
FNE1	20	4	1	0.7	0	0	74.3	72.8 ± 0.05	5.60
FNE2									
(a)	20	4	1	0.7	0.2	0	74.1	112.7 ± 0.12	5.66
(b)	20	4	1	0.7	0.4	0	73.9	133.5 ± 0.12	5.64
(c)	20	4	1	0.7	0.6	0	73.7	104.5 ± 0.13	5.53
(d)	20	4	1	0.7	0.8	0	73.5	90.9 ± 0.10	5.47
(e)	20	4	1	0.7	1.0	0	73.3	113.6 ± 0.11	5.36
FNE3									
(a)	20	4	1	0.7	0.2	1	73.1	115.2 ± 0.13	5.65
(b)	20	4	1	0.7	0.4	1	72.9	144.8 ± 0.08	5.61
(c)	20	4	1	0.7	0.6	1	72.7	94.9 ± 0.11	5.55
(d)	20	4	1	0.7	0.8	1	72.5	98.6 ± 0.10	5.54
(e)	20	4	1	0.7	1.0	1	72.3	99.8 ± 0.15	5.40
FNE4									
(a)	20	4	1	0.7	0.2	2	72.1	103.5 ± 0.14	5.73
(b)	20	4	1	0.7	0.4	2	71.9	144.3 ± 0.09	5.70
(c)	20	4	1	0.7	0.6	2	71.7	114.7 ± 0.15	5.66
(d)	20	4	1	0.7	0.8	2	71.5	91.6 ± 0.08	5.68
(e)	20	4	1	0.7	1.0	2	71.3	99.0 ± 0.13	5.34
FNE5									
(a)	20	4	1	0.7	0.2	3	71.1	110.8 ± 0.11	5.68
(b)	20	4	1	0.7	0.4	3	70.9	155.0 ± 0.12	5.64
(c)	20	4	1	0.7	0.6	3	70.7	124.6 ± 0.14	5.58
(d)	20	4	1	0.7	0.8	3	70.5	103.7 ± 0.13	5.45
(e)	20	4	1	0.7	1.0	3	70.3	100.7 ± 0.13	5.40

**Table 4:** Power law model parameters for selected FNE formulations.

Formulation	Components concentration (%, w/w)		Consistency coefficients, *K* (Pa s^*n*^)	Flow behavior indices, *n*	Regression coefficients, *R* ^2^
	Xanthan gum	Beeswax			
FNE3					
(b)	0.4	1	3.173	0.202	0.983
(c)	0.6	1	7.305	0.144	0.976
(d)	0.8	1	9.438	0.137	0.988
(e)	1	1	12.49	0.147	0.99
FNE4					
(b)	0.4	2	4.318	0.217	0.981
(c)	0.6	2	8.742	0.104	0.98
(d)	0.8	2	10.32	0.137	0.971
(e)	1	2	15.78	0.132	0.984
FNE5					
(b)	0.4	3	5.247	0.19	0.986
(c)	0.6	3	8.886	0.143	0.981
(d)	0.8	3	11.02	0.141	0.98
(e)	1	3	21.32	0.095	0.974

**Table 5:** Temperature-dependent activation parameter for PKOEs/Tween 80 : Span 80/water systems.

Formulation	Δ*H *(kJ mol^−1^)				Δ*G *(kJ mol^−1^)				Δ*S* (J K^−1^ mol^−1^)			
	Temperature, K				Temperature, K				Temperature, K			
	298	303	308	313	298	303	308	313	298	303	308	313
Pre-F	23.8	16.2	8.0	−0.7	38.8	38.4	38.1	37.9	−50.5	−75.3	−101.6	−129.1
FNE1	96.1	67.3	36.4	3.3	39.9	38.7	38.4	38.3	188.6	92.0	−10.7	−117.8
FNE2												
(a)	47.6	29.3	9.8	−11.1	57.6	56.5	56.0	55.5	−33.8	−92.9	−156.1	−221.6
(b)	98.9	80.2	59.9	38.3	60.9	59.8	58.5	58.1	127.5	63.7	−1.7	−72.7
(c)	107.5	82.1	54.9	25.7	62.9	61.7	60.9	60.6	149.6	64.1	−26.3	−121.3
(d)	108.2	69.2	27.3	−17.4	64.0	62.5	62.2	62.0	148.3	18.5	−120.0	−263.8
(e)	81.7	60.8	38.3	14.3	64.7	63.9	63.3	63.3	57.1	−13.7	−88.0	−166.6
FNE3												
(a)	52.8	59.2	65.9	72.9	57.8	57.1	56.3	55.4	−16.8	3.7	25.0	46.9
(b)	91.7	71.9	50.6	27.9	61.3	60.0	59.5	59.0	102.0	36.0	−35.4	−109.0
(c)	96.5	72.3	46.3	18.5	63.2	61.8	61.2	60.7	111.8	31.1	−55.0	−144.7
(d)	107.0	77.0	44.9	10.5	64.1	62.8	62.2	62.1	144.0	43.3	−63.1	−174.8
(e)	91.0	65.8	38.8	9.9	65.0	64.1	63.5	63.5	87.0	2.2	−87.1	−181.2
FNE4												
(a)	63.3	64.7	66.0	67.4	57.9	57.2	56.4	55.7	18.0	21.5	25.2	28.4
(b)	69.0	52.2	34.2	15.0	61.4	60.4	59.7	59.3	25.3	−30.4	−89.1	−151.0
(c)	81.2	58.8	34.7	9.0	63.5	62.2	61.6	61.1	59.5	−14.7	−94.1	−176.4
(d)	98.1	73.9	48.0	20.2	64.3	63.1	62.6	62.3	113.4	32.0	−54.3	−144.5
(e)	78.3	60.4	41.1	20.4	65.2	64.3	63.8	63.6	44.2	−16.6	−80.7	−148.2
FNE5												
(a)	68.0	61.1	53.7	45.7	58.2	57.5	56.7	56.2	32.7	8.8	−15.8	−42.7
(b)	83.7	62.1	38.9	14.1	61.9	60.6	60.0	59.5	73.1	1.6	−75.2	−154.7
(c)	95.2	68.7	40.2	9.8	63.9	62.6	62.1	61.8	105.3	16.6	−77.6	−176.1
(d)	81.7	60.0	36.8	11.9	64.4	63.5	63.3	63.1	57.9	−14.9	−93.0	−173.8
(e)	73.5	56.1	37.5	17.5	65.2	64.4	63.9	63.7	27.6	−31.0	−92.7	−157.8

**Table 6:** Accelerated stability assessment of the Pre-F and FNEs.

Formulation	Centrifugation test at 4000 rpm for 15 min	298 K	318 K	Freeze-thaw cycles
Pre-F	*✗*	—	—	*✗*
FNE1	*✗*	—	—	*✗*
FNE2				
(a)	*✓*	*✓*	*✗*	*✓*
(b)	*✓*	*✓*	*✗*	*✓*
(c)	*✓*	*✓*	*✓*	*✓*
(d)	*✓*	*✓*	*✓*	*✓*
(e)	*✓*	*✓*	*✓*	*✓*
FNE3				
(a)	*✓*	*✓*	*✗*	*✓*
(b)	*✓*	*✓*	*✗*	*✓*
(c)	*✓*	*✓*	*✓*	*✓*
(d)	*✓*	*✓*	*✓*	*✓*
(e)	*✓*	*✓*	*✓*	*✓*
FNE4				
(a)	*✓*	*✓*	*✗*	*✓*
(b)	*✓*	*✓*	*✗*	*✓*
(c)	*✓*	*✓*	*✓*	*✓*
(d)	*✓*	*✓*	*✓*	*✓*
(e)	*✓*	*✓*	*✓*	*✓*
FNE5				
(a)	*✓*	*✓*	*✗*	*✓*
(b)	*✓*	*✓*	*✗*	*✓*
(c)	*✓*	*✓*	*✓*	*✓*
(d)	*✓*	*✓*	*✓*	*✓*
(e)	*✓*	*✓*	*✓*	*✓*

Note: *✓*,stable (without phase separation); *✗*, unstable (with phase separation).
